# Risk of hematological malignancies associated with magnetic fields exposure from power lines: a case-control study in two municipalities of northern Italy

**DOI:** 10.1186/1476-069X-9-16

**Published:** 2010-03-30

**Authors:** Carlotta Malagoli, Sara Fabbi, Sergio Teggi, Mariagiulia Calzari, Maurizio Poli, Elena Ballotti, Barbara Notari, Maurizio Bruni, Giovanni Palazzi, Paolo Paolucci, Marco Vinceti

**Affiliations:** 1 CREAGEN - Environmental, Genetic and Nutritional Epidemiology Research Center, Department of Public Health Sciences, University of Modena and Reggio Emilia, via Campi 287, 41125 Modena, Italy; 2 LARMA - Laboratory of Environmental Analysis, Surveying and Environmental Monitoring, Department of Mechanical and Civil Engineering, University of Modena and Reggio Emilia, via Vignolese 905, 41125 Modena, Italy; 3 Local Health Unit of Reggio Emilia, via Amendola 2, 42122 Reggio Emilia, Italy; 4 ARPA - Emilia Romagna Environmental Protection Agency, section of Reggio Emilia, via Amendola 2, 42122 Reggio Emilia, Italy; 5 ARPA - Emilia Romagna Environmental Protection Agency, section of Modena, via Fontanelli 23, 41121 Modena, Italy; 6 Department of Mother and Child, University of Modena and Reggio Emilia, via del Pozzo 71, 41124 Modena, Italy

## Abstract

**Background:**

Some epidemiologic studies have suggested an association between electromagnetic field exposure induced by high voltage power lines and childhood leukemia, but null results have also been yielded and the possibility of bias due to unmeasured confounders has been suggested.

**Methods:**

We studied this relation in the Modena and Reggio Emilia municipalities of northern Italy, identifying the corridors along high voltage power lines with calculated magnetic field intensity in the 0.1-<0.2, 0.2-<0.4, and ≥ 0.4 microTesla ranges. We identified 64 cases of newly-diagnosed hematological malignancies in children aged <14 within these municipalities from 1986 to 2007, and we sampled four matched controls for each case, collecting information on historical residence and parental socioeconomic status of these subjects.

**Results:**

Relative risk of leukemia associated with antecedent residence in the area with exposure ≥ 0.1 microTesla was 3.2 (6.7 adjusting for socioeconomic status), but this estimate was statistically very unstable, its 95% confidence interval being 0.4-23.4, and no indication of a dose-response relation emerged. Relative risk for acute lymphoblastic leukemia was 5.3 (95% confidence interval 0.7-43.5), while there was no increased risk for the other hematological malignancies.

**Conclusions:**

Though the number of exposed children in this study was too low to allow firm conclusions, results were more suggestive of an excess risk of leukemia among exposed children than of a null relation.

## Background

Since the original observation by Wertheimer and Leeper in 1979 [1], several epidemiologic studies have suggested an association between magnetic fields exposure [2,3], such as that induced by residence near high voltage power lines, and childhood leukemia, but some investigations yielded null results and the possibility of bias induced by unmeasured confounders or inappropriate exposure assessment in the former studies has been suggested [4-6]. Moreover, no consensus exists about the cutpoints of magnetic field exposure above which leukemia risk might actually increase, and little evidence is available on a relation with risk of other hematological malignancies in childhood, suggesting the need for new methodological approaches to this issue of primary public health importance [7,8].

We investigated the possible association between magnetic fields exposure and risk of leukemia and other hematological cancers in the pediatric population of two municipalities in northern Italy, taking into consideration parental socioeconomic status as a potential confounder.

## Methods

### Study population

We attempted to identify all cases of hematological cancers newly-diagnosed during the 1986-2007 period in children while residing in the two northern Italy municipalities of Modena e Reggio Emilia (around 180,000 and 160,000 inhabitants in 2007, respectively). To do so, we used the nation-wide hospital-based registry of childhood malignancies managed by the Associazione Italiana Ematologia Oncologia Pediatrica (AIEOP) [9]. The AIEOP Registry, operating since 1985 and based on collaboration with 54 specialized hospital centers in Italy belonging to this network, has to date registered 35,256 newly diagnosed patients up to the age of 14. However, the physicians were aware that patients aged 14 were likely to be referred to the Hematological Departments usually taking care of adults: we therefore decided to limit the analysis to the subjects aged 0-13 to warrant a population-based design in patients' selection, removing two subjects from the AIEOP database (one with acute acute lymphoblastic leukemia and the other with non-Hodgkin's lymphoma) and consequently from further analysis. We randomly selected for each case four controls from the general population, by extracting these referents from the municipal databases of residents matched for year of birth, sex and municipality of residence to each case, and using historical databases of residents according to the year of diagnosis. We then reconstructed with the directories of the General Registry Offices the residential history of cases and controls, starting from date of birth, if the child had always resided in the municipality, or from the date of immigration into the municipality when appropriate. In case of uncertain data, we contacted family doctors, or when necessary the families directly. Information about paternal and maternal educational attainment level and paternal income was also collected from the General Registry Offices and through web-available resources.

### Exposure assessment

We identified the high voltage power lines (≥ 132 kilovolts) located during the 1986-2007 period in the municipal territories of Modena and Reggio Emilia, whose code, average current intensity and voltage are reported in table 1. We then calculated magnetic field induction in the proximity of these lines using two models, CAMPI http://www.ifac.cnr.it/pcemni/manuale.pdf and EFC400 http://www.fgeu.de/html/demos.htm, to define the distance at which, at a height of 8 m, the magnetic fields intensity cutpoints of 0.1, 0.2 and 0.4 microTesla (μT) occurred. CAMPI is a freeware software simulation package developed by Andreuccetti at the Florence National Research Council Applied Physics Institute, to predict the intensity of the magnetic flux density (*B*) generated by power lines. The program is based on a 2D model, where the power line is represented with a set of straight, horizontal, infinite and parallel conductors. The ground is considered as not affecting the magnetic field and interactions with obstacles (towers, trees or buildings) placed along the line are also neglected. Calculation of the *B *of each conductor is based on the application of the Biot-Savart formula and therefore, resulting directly proportional to the intensity of the current flowing in the conductor and inversely proportional to the distance between the conductor and the point where the field is computed. In the few cases of vicinity between two or more power lines, we used the alternative model generated by commercial EFC400 software to estimate magnetic fields distribution in these complex situations. EFC-400 is a commercial software simulation package developed by FGEU mbH (ForschungsGesellschaft für Energie und Umwelttechnologie mbH, Berlin) to predict the intensity of the magnetic flux density generated by power lines in a 3D model. The algorithm used is the same as the CAMPI model, but the conductors are divided into a certain numbers of portions (up to 20) which are considered as single sources of magnetic field, to calculate overall resulting *B *in prediction points. In this way EFC-400 is able to simulate complex situations, like closing, crossing, curved and finite electric lines. Both models have been validated in a survey which confirmed that both models carefully predicted the magnetic field distribution detected through on-site measurements around a high voltage (132 kilovolts) power line in the bordering Bologna province [10]. For both CAMPI and EFC400 models, we considered the characteristics and the disposition of conductors of each power line, and we used the average current run in the power lines during 2001, which is the earliest year datum made available by the regional environmental protection agency, ARPA, due to mandatory reporting of currents by the electric companies in compliance with the Emilia-Romagna Region rule 'Direttiva 197/2001'. We thus identified three 'exposed' corridors (0.1 ≤ *B*<0.2, 0.2 ≤ *B*<0.4, *B *≥ 0.4 μT) surrounding the power lines, whose distance from the conductors is reported in table 1 along which the main technical characteristics of the power lines crossing the two study municipalities. Historical analysis of current consumption and requirements over the 1986-2007 period according to national electric company data http://www.terna.it/default/home/sistema_elettrico/statistiche.aspx indicated that the currents run in power lines in the Modena and Reggio Emilia provinces increased over time of about 3% on a yearly basis, and that the 2001 values were respectively 13.5% and 13.3% higher than the average values of the whole period.

**Table 1:** Distance of exposure corridors from conductors (at calculated magnetic fields cutpoints of 0.1, 0.2 and 0.4 μT along the high voltage power lines in Modena and Reggio Emilia municipalities

Modena municipality					
**Line Code**	**Voltage (kilovolts)**	**Electric current (amperes, average at 2001)**	**Distance from power line (m)**		
			
			**0.1 μT**	**0.2 μT**	**0.4 μT**

175	132	415	94	67	48
176	132	128	52	37	27
176/686	132	238	88	62	44
686	132	238	59	42	30
600	132	378	74	53	38
175/600	132	415	108	77	54
614	132	110	61	43	30
615	132	95	40	28	20
625	132	80	35	25	18
634	132	121	45	32	22
638	132	31	23	16	11
688	132	360	88	63	45
631/632	132	94	52	36	26
300	380	273	84	60	43
320	380	414	104	73	52
395	380	285	86	61	44
300/320	380	414	89	67	51
BO 017	132	10	14	10	7
BO 018	132	14	15	11	8

**Reggio Emilia municipality**					

**Line Code**	**Voltage (kilovolts)**	**Electric current (amperes, average at 2001)**	**Distance from power line (m)**		
			
			0.1 μT	0.2 μT	0.4 μT

103	132	21	18	13	9
660	132	88	35	25	18
698	132	158	45	32	23
315	380	404	99	72	51
683	132	107	39	28	20
104	132	91	44	32	23
642	132	63	29	20	14
656	132	41	30	22	16
659	132	170	60	42	30
668	132	34	20	14	10
677	132	60	29	20	15
685	132	233	85	60	43
RFI-BO PR	132	25	20	14	10

We included the exposure corridors in a Geographical Information System (GIS), using ARC-GIS software (version 9.2, ESRI, Redlands, CA 2006), and we geocoded the historical residences of all study subjects in this GIS database. To do so, we identified the centroid of the exact address of each subject's residence on the ARC-GIS municipal maps: when the building was not found in the files of Modena and Reggio Emilia municipalities, as occurred in 23 locations, we directly measured the coordinates on site using a global positioning system device (Garmin GPSmap 60CSx, Garmin Int. Corp., Olathe, KS). We eventually defined study subjects as 'exposed' if they had resided for at least six months in one of the exposed corridors, providing that the power lines were actually in operation. However, the six months prior to the date of diagnosis for cases and the corresponding period for ther matched controls were excluded from this analysis.

### Data analysis

We calculated the relative risks (RR) with their 95% confidence interval (CI) of hematological cancers and more specifically of all leukemias, acute lymphoblastic leukemia and non-leukemic malignancies associated with antecedent exposure to magnetic fields by calculating the odds ratio of the disease in conditional and unconditional logistic regression models, using the STATA statistical package (Stata version 10.1 for Windows, Stata Corporation, TX 2009). We also repeated the analysis adjusting for three potential confounders, paternal educational attainment level, maternal educational attainment level, and paternal income. Statistical precision of the risk estimates was evaluated by computing their 95% confidence limits.

## Results

We identified 64 cases of childhood hematological malignancies newly-diagnosed during the study period (table 2), to whom we matched 256 population controls randomly drawn from the general populations. Of these diseases, 46 were cases of leukemia, 36 of which were of the acute lymphoblastic leukemia subtype, and the remaining 18 cases were included in three different nosological entities. Data about parental socioeconomic status of case and control children are reported in table 3.

**Table 2:** Classification of childhood hematological malignancies diagnosed in the Modena and Reggio Emilia municipal populations from 1986 to 2007

Diagnosis	ICD-9^a^	n	(%)
All malignant neoplasms of lymphatic and hematopoietic tissue	200-208	64	(100.0)
Lymphosarcoma and reticulosarcoma and other specified malignant tumors of lymphatic tissue			
Lymphosarcoma	200.1	6	(9.3)
Burkitt's tumor or lymphoma	200.2	2	(3.1)
Hodgkin's disease			
Lymphocytic-histiocytic predominance	201.4	1	(1.6)
Nodular sclerosis	201.5	1	(1.6)
Mixed cellularity	201.6	1	(1.6)
Hodgkin's disease, unspecified	201.9	2	(3.1)
Other malignant neoplasms of lymphoid and histiocytic tissue			
Letterer-Siwe disease	202.5	5	(7.8)
Lymphoid leukemia			
Acute lymphoid leukemia	204.0	36	(56.2)
Chronic lymphoid leukemia	204.1	1	(1.6)
Unspecified lymphoid leukemia	204.9	1	(1.6)
Myeloid leukemia			
Acute myeloid leukemia	205.0	3	(4.7)
Chronic myeloid leukemia	205.1	1	(1.6)
Unspecified myeloid leukemia	205.9	1	(1.6)
Monocytic leukemia			
Acute monocytic leukemia	206.0	3	(4.7)

^a ^International Classification of Diseases, 9^th^edition

**Table 3:** Parental socioeconomic status of children with newly-diagnosed hematological disease during the 1986-2007 period and their matched controls, Modena and Reggio Emilia municipalities, northern Italy

	All hematological malignancies		All leukemias		Acute lymphoblastic leukemia	
			
	Cases (n = 64) n (%)	Controls (n = 256) n (%)	Cases (n = 46) n (%)	Controls (n = 184) n (%)	Cases (n = 36) n (%)	Controls (n = 144) n (%)
*Residence*						
Modena	30(46.9)	120(46.9)	21(45.7)	84(45.7)	17(47.2)	68(47.2)
Reggio Emilia	34(53.1)	136(53.1)	25(54.3)	100(54.3)	19(52.8)	76(52.8)
*Gender*						
Male	35(54.7)	140(54.7)	24(52.2)	96(52.2)	19(52.8)	76(52.8)
Female	29(45.3)	116(45.3)	22(47.8)	88(47.8)	17(47.2)	68(47.2)
*Paternal income*^*a*^						
0	0(0.0)	11(4.3)	0(0.0)	8(4.4)	0(0.0)	7(4.9)
>0-8	7(10.9)	23(9.0)	5(10.9)	17(9.2)	5(13.9)	13(9.0)
9-15	4(6.2)	26(10.2)	4(8.7)	18(9.8)	3(8.3)	14(9.7)
16-25	14(21.9)	50(19.5)	9(19.6)	35(19.0)	6(16.7)	27(18.7)
26-40	8(12.5)	45(17.6)	7(15.2)	31(16.9)	4(11.1)	27(18.7)
41-100	10(15.6)	47(18.4)	7(15.2)	32(17.4)	6(16.7)	26(18.1)
>100	1(1.6)	5(1.9)	1(2.2)	5(2.7)	1(2.8)	5(3.5)
Unknown	20(31.2)	49(19.1)	13(28.2)	38(20.6)	11(30.6)	25(17.4)
*Paternal educational attainment*						
<Primary school	0(0.0)	3(1.2)	0(0.0)	2(1.0)	0(0.0)	2(1.4)
Primary school	6(9.4)	20(7.8)	3(6.5)	16(8.7)	3(8.3)	14(9.7)
Middle school	21(32.8)	81(31.6)	14(30.4)	53(28.8)	11(30.6)	38(26.4)
High school	12(18.7)	86(33.6)	11(24.0)	63(34.3)	9(25.0)	51(35.4)
University	11(17.2)	40(15.6)	8(17.4)	30(16.3)	5(13.9)	25(17.4)
Unknown	14(21.9)	26(10.2)	10(21.7)	20(10.9)	8(22.2)	14(9.7)
*Maternal educational attainment*						
<Primary school	0(0.0)	2(1.8)	0(0.0)	0(0.0)	0(0.0)	0(0.0)
Primary school	4(6.2)	26(10.2)	3(6.5)	19(10.3)	3(8.3)	16(11.1)
Middle school	23(35.9)	98(38.3)	17(37.0)	73(39.7)	17(47.2)	55(38.3)
High school	17(26.6)	80(31.2)	13(28.3)	59(32.0)	7(19.4)	45(31.2)
University	8(12.5)	30(11.7)	7(15.2)	18(9.8)	4(11.1)	16(11.1)
Unknown	12(18.7)	20(7.8)	6(13.0)	15(8.2)	5(13.9)	12(8.3)

^a ^Thousands of euros/year 2005

Two cases (all affected by acute lymphoblastic leukemia), diagnosed in 2003 and in 2006, and five controls had been exposed to electromagnetic fields from power lines, since they had resided for more than six months in one of the exposed corridors. None of these subjects had resided for less than six months in one of the three exposed corridors before diagnosis, nor had been residing in more than one of these corridors. All of the exposed children had been continuously residing in one of these corridors for more than one year, starting from 13 months up to 6.5 years. One case and three control children had been residing in the corridor with the 0.1-<0.2 μT range of exposure, while the other case and the remaining two controls resided in the corridor with exposure ≥ 0.4 μT.

The risk of hematological malignancies associated with antecedent exposure to magnetic fields from power lines was 1.7 (95% CI 0.3-9.4) in the unadjusted analysis and 2.4 (95% CI 0.4-15.0) after adjusting for socioeconomic status (table 4). Limiting the analysis to leukemia cases and matched controls, the relative risk associated to magnetic field exposure was 3.2 (95% CI 0.4-23.4), which further increased to 6.7 (95% CI 0.6-78.3) after adjusting for socioeconomic status indicators. Corresponding figures for the acute lymphoblastic leukemia were 6.0 (95% CI 0.5-69.5) for crude analysis and 5.3 (95% CI 0.7-43.5) for the multivariate estimate, which, however, could only be computed in an unconditional logistic regression model. Conversely, we could not obtain a valid RR estimate for the hematological malignancies other than leukemia overall considered, a category for which we did not identify any exposed case.

**Table 4:** Relative risk (RR) of childhood hematological disease associated with magnetic field exposure from power lines ≥ 0.1 μT and ≥ 0.4 μT in Modena and Reggio Emilia municipalities, 1986-2007^a^

Disease		< 0.1 μT		≥ 0.1 μT			≥ 0.4 μT		
		
		cases/controls	RR	cases/controls	RR	95% CI^b^	cases/controls	RR	95% CI^b^
All hematological malignancies	Crude	62/251	1.00	2/5	1.7	0.3-9.4	1/2	2.4	0.1-42.6
	Adjusted^c^	36/184	1.00	2/5	2.4	0.4-15.0	1/2	2.8^d^	0.2-34.3^d^
All leukemias	Crude	44/181	1.00	2/3	3.2	0.4-23.4	1/2	2.4	0.1-42.6
	Adjusted^c^	27/129	1.00	2/3	6.7	0.6-78.3	1/2	2.1^d^	0.2-26.2^d^
Acute lymphoblastic leukemia	Crude	34/142	1.00	2/2	6.0	0.5-69.5	1/2	2.4	0.1-42.6
	Adjusted^c^	20/106	1.00	2/2	5.3^d^	0.7-43.5^d^	1/2	2.3^d^	0.2-29.1^d^
Hematological malignancies other than leukemia	Crude	18/70	1.00	0/2	-^e^	-^e^	0/0	-^e^	-^e^
	Adjusted^c^	9/55	1.00	0/2	-^e^	-^e^	0/0	-^e^	-^e^

^a^Relative risk estimates calculated with conditional logistic regression, using exposure category < 0.1 μT as reference category

^b^95% confidence interval

^c^Adjusted for paternal educational attainment and income and for maternal educational attainment

^d^Risk estimates computed with unconditional logistic regression, adjusting for age, sex, municipality, paternal educational attainment and income, and maternal educational attainment

^e^No statistically valid estimates yielded by both conditional and unconditional logistic regression

Limiting the analysis of exposed children to those with highest exposure, i.e. ≥ 0.4 μT, RR, overall risk for the malignancies considered showed a slight increase compared with the previous analysis, but this was not true for leukemia or for acute lymphoblastic leukemia alone.

## Discussion

We must acknowledge a major limitation of this study and which limits interpretation of our data, i.e. the low number of cases and in particular the limited number of exposed cases, thus yielding statistically unstable risk estimates and hampering the prospect of detecting small changes in risk and limiting the chance to assess possible dose-response relations. Another limitation of the study is the use of calculated magnetic fields based on power lines configuration and not on direct measurements of exposure. However, previous studies have shown that, particularly for children, this methodology tends to be substantially adequate in reflecting actual exposure to power lines-induced magnetic fields, and that this source of exposure tends to exceed the contribution of other sources such as home appliances [11], particularly in the case of high voltage transmission lines [12]. Moreover, we collected in the present study the entire residential history of the study subjects, and therefore we were able to estimate their historical exposure status, while assessments based on single measurements (generally for cases of children's residence at diagnosis) might have led to substantial misclassification of exposure [13]. Finally, we obtained information from the Modena and Reggio Emilia municipalities about location of primary schools with reference to magnetic fields from power lines, to investigate this potential source of confounding. None of the schools were located in an area with magnetic field intensity ≥ 0.1 μT in Modena or ≥ 0.2 μT in Reggio Emilia (data were unavailable for the 0.1-<0.2 range in Reggio Emilia).

In this study we used a disease registry (the AIEOP Registry) which was hospital-based and not population-based, but we consider it unlikely that this feature of the study biased the study results. In fact, all children affected by hematological malignancies are expected to be referred to hospital and to highly-specialized centers in particular, all of which are included in the AIEOP network. Moreover, we verified the completeness of this registry for hematological cancers in the Reggio Emilia municipality by comparing its database with information collected in loco from several data sources (hospital discharge registry, drug prescriptions, family doctors, death certificates) [14]. All but one case was reported to the study registry, which in turn was an independent source for only one additional case.

To assess exposure of study subjects through the period 1986-2007 in this investigation, we defined exposure corridors through calculation of magnetic fields based on average currents run in the year 2001, the earliest available, which were nearly 13% higher than the average current in the whole study period, thus raising the risk of some misclassification of exposure. However, in this investigation, the two cases found to be exposed received their diagnosis after 2001, thus suggesting that the methodology we used did not overestimate exposure status of these children and did not spuriously increase the RR estimates in exposed subjects.

In our study population, we found a prevalence of exposure to magnetic fields ≥ 0.4 μT of 0.94%, higher than the available figures for the Italian population of 0.20-0.35% [8], but this is likely due not only to our limited sample size and the uncertainties of the national estimates (which were calculated in the 1990s when currents run in power lines were still not officially available), but also to the urban, densely populated setting examined in the present investigation.

This study has some strengths compared with other investigations carried out in this field. First, the entire residential history of study subjects was available, a characteristic which made it possible to investigate the lifetime exposure and to account for adequate induction and latent periods from exposure to the disease. Moreover, we could acquire all study data (residential histories and socioeconomic information) without directly contacting the families, thus avoiding the risk of recall and selection bias due to refusal to participate, and consequently the likelihood of generating inaccurate information. We were also able to adjust the analysis for three indicators of socioeconomic status including paternal income, thus reducing the risk of substantial confounding, though no obvious relation between socioeconomic status and leukemia risk appears to exist [15], and in the present study risk estimates did not substantially change after adjusting for these indicators. Finally, we selected the controls on the basis of the historical databases of municipal residents matched to years of diagnosis of cases, different to previous case-control studies where the referents were sampled from a single, recently available directory of residents, despite the long period of time during which disease diagnoses occurred in cases (even in the order of 15-20 years). In such investigations, general population residential distribution might have substantially changed over time with reference to proximity to power lines, thus biasing exposure status of controls.

Overall, study results showed an excess risk of leukemia, but not of other hematological malignancies, in children exposed to magnetic fields generated from power lines, and this finding appears in line with most studies carried out on this topic [6,8], though the statistical instability of risk estimates and the lack of dose-response relations do not allow for the alternative hypothesis of no effect on disease risk of magnetic fields exposure to be entirely ruled out. In fact, an increased childhood leukemia risk in the proximity of high voltage power lines was detected not only in a recent large case-control investigation [16] but also in two smaller studies carried out in Italy [17] and in Tasmania (courtesy of Ray Lowenthal, Deirdre Tuck and Isabelle Bray, unpublished data), suggesting a homogeneous trend across different populations. It should also be noted that recent laboratory studies have increased the biological plausibility of adverse health effects of extremely low frequency magnetic fields [18,19].

## Conclusions

Findings of the present study, which are consistent with those of recent case-control investigations independently of their characteristics and sample size [6-8], appear to support the hypothesis that magnetic fields exposure increases the risk of childhood leukemia, though likely representing a risk factor for a large minority of cases, as expected on the basis of epidemiologic evidence [20].

## Abbreviations

GIS: Geographical Information System; ARPA: Agenzia Regionale per la Protezione Ambientale; μT: microTesla; *B*: magnetic flux density; AIEOP: Associazione Italiana Ematologia Oncologia Pediatrica; ICD-9: International Classification of Diseases, 9^th ^Edition; RR: relative risk; CI: confidence interval.

## Competing interests

The authors declare that they have no competing interests.

## Authors' contributions

CM and MV conceived and coordinated the study, performed data analysis and drafted the manuscript; SF and ST designed and coordinated the GIS database; MP, EB, BN and MB collected information about power lines configuration and calculated magnetic fields around the lines; MC, GP and PP identified the incident cases. All authors have read and approved the final manuscript.
